# The Importance of Basal Cell Carcinoma Risk Stratification and Potential Future Pathways

**DOI:** 10.2196/50309

**Published:** 2023-10-30

**Authors:** Sharad Paul, Allanah Knight

**Affiliations:** 1 Auckland University of Technology Auckland New Zealand; 2 Skin Surgery Clinic Auckland New Zealand

**Keywords:** skin cancer, BCC, basal cell carcinoma, dermatology, histology, cancer, tumour markers, angiogenic agents, angiogenic, carcinoma, skin, risk assessment, management, surgery, angiogenic marker, markers, immunohistochemistry

## Abstract

**Background:**

Basal cell carcinoma (BCC) is the most common human cancer. Although there are surgical and topical treatments available, surgery remains the mainstay of treatment, leading to higher costs. What is needed is an accurate risk assessment of BCC so that treatments can be planned in a patient-centered manner.

**Objective:**

In this study, we will review the literature about guidelines for the management of BCC and analyze the potential indicators of high-risk BCC. Using this risk assessment approach, we will propose pathways that will be able to optimize treatments more efficiently.

**Methods:**

This paper presents a perspective from a skin cancer expert and clinic involved in the treatment of both simple and complex cases of BCC. It addresses the key challenges associated with accurate risk stratification prior to any treatment or procedure. Different immunohistochemical and angiogenic markers for high-risk BCC were reviewed in this study.

**Results:**

The expression of interleukin-6, vascular endothelial growth factor, and mast cells within BCC correlates with its aggressiveness. Other immunohistochemical markers, such as Cyclin D1 and Bcl-2, also play a significant role—Cyclin D1 is higher in the aggressive BCC, while Bcl-2 is lower in the aggressive BCC, compared to the nonaggressive variants.

**Conclusions:**

Based on our research, we will conclude that using immunohistochemical and angiogenic markers for risk assessment and stratification of BCC can help optimize treatment, ensuring that surgical procedures are used only when necessary.

## Introduction

### Basal Cell Carcinoma

Basal cell carcinoma (BCC) is the most common cancer in humans, with the estimated incidence having risen by 20% and 80% over the past 30 years. As a result, it is expected that 1 in 5 Americans may develop BCC in their lifetime [1]. Mutagenesis of p53 appears preferentially in the aggressive variants of BCC, and BCC in sun-exposed and sun-protected sites seem to have different biology and morphology [2].

Excisional surgery is the mainstay of treatment, but the biology of BCC always entails a recurrence rate, with reports suggesting a recurrence rate of 2%-8% at 5 years following standard surgery [3] and 3%-4% following Mohs micrographic surgery [4]. This may be because the biological transformation of BCC tends to occur at the base and edges of the lesion [5]. Given this recurrence rate, it is even more important to differentiate between high- and low-risk BCC (aggressive and indolent variants) so surgical and nonsurgical options can be used appropriately.

### High- and Low-Risk BCC

**Figure 1:**
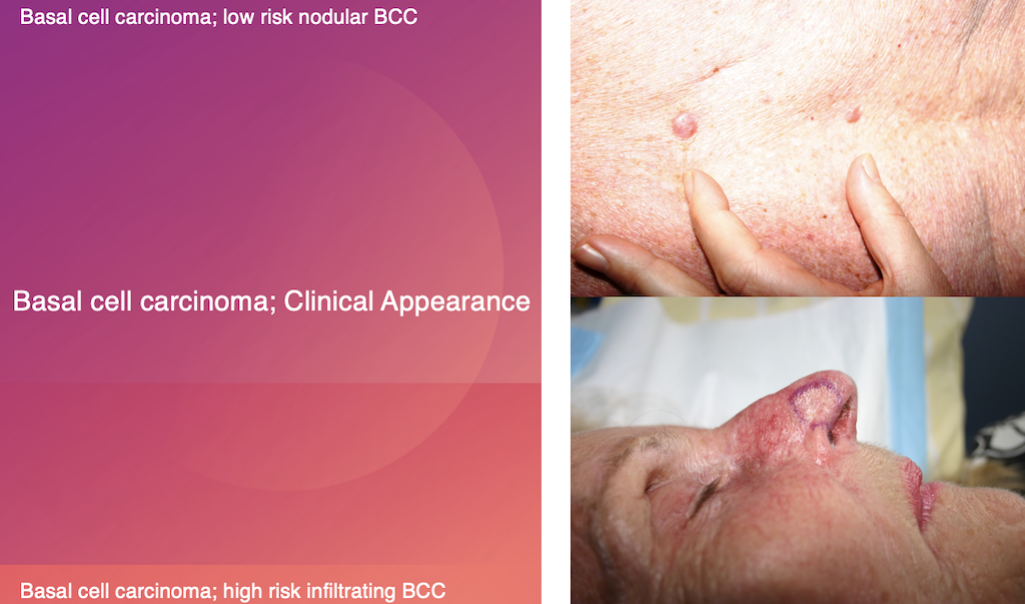
Basal cell carcinoma (clinical appearance); low risk nodular BCC and high risk infiltrating BCC.

**Figure 2:**
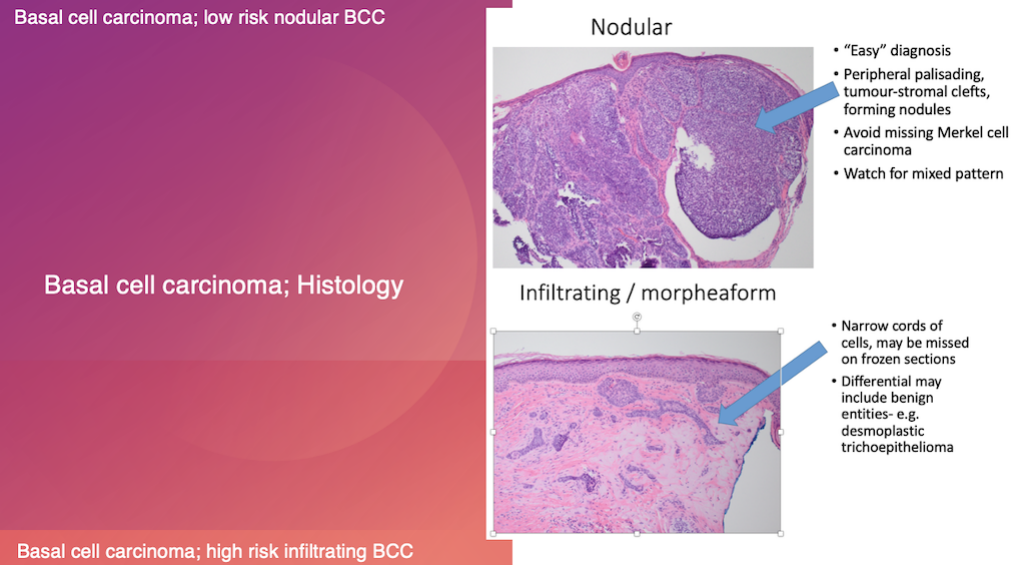
Basal cell carcinoma (histology); low risk nodular BCC and high risk infiltrating BCC.

**Figure 3:**
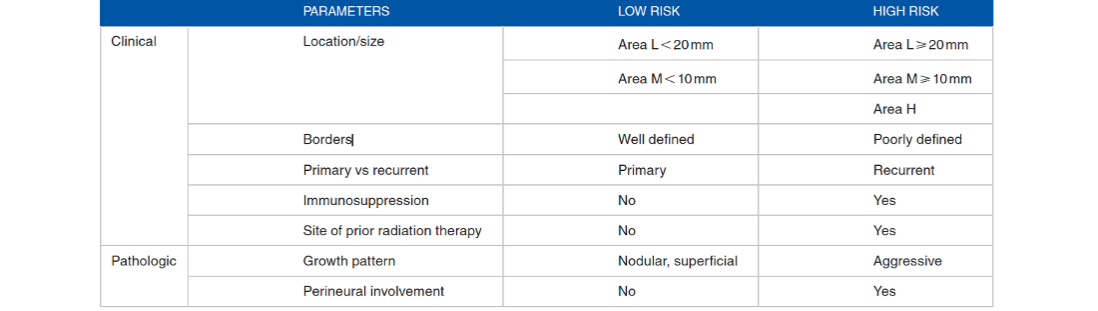
Clinical and pathological parameters to determine basal cell carcinoma (BBC) risk (adapted from Badash et al [9]).

Superficial and nodular BCCs are considered indolent, whereas infiltrative BCC, metatypical BCC, and morpheaform or sclerosing BCC are considered aggressive [6]. Some clinical presentations and histopathology insights are illustrated in Figures 1 and 2. It is well known that micronodular BCC has a higher recurrence rate because tumor extensions are more difficult to assess both clinically and histologically [7]. There are also clinical and pathological parameters used to assess the risk of BCC (Figure 3 [8]) [9].

### Treatment of BCC

The aims of surgical treatment for BCC are fourfold: to remove both the clinically visible tumor and its microscopic extensions into the surrounding normal-appearing skin, to prevent recurrence in the future, to avoid any functional impairment from the surgery, and to provide the best possible cosmetic outcomes for the patient. Surgical approaches to BCC, including recommended margins for high- and low-risk BCC, vary across different geographical locations, and they are summarized in Table 1.

One of the major issues related to the management of BCC has been the cost of treatment, which has raised concerns among health authorities. In Australia, the total cost of keratinocyte skin cancers (BCC and squamous cell carcinoma) in 2021 was US $426.2 million, and in New Zealand, it was US $129.4 million [10]. Even more than a decade ago, the costs of nonmelanoma treatment for skin cancer in the United States were substantial. In a study conducted between 2007 and 2011 [11], 4.9 million US adults were treated for skin cancer per annum, with an annual treatment cost of US $8.1 billion. Authorities have also shown concerns about doctors’ overtreatment. The US Department of Justice and the Centers for Medicare and Medicaid Services have investigated dermatologists for alleged Medicare fraud related to overuse of Mohs surgery [12]. Therefore, it is crucial for clinicians to be able to identify high-risk and low-risk BCC before any expensive surgery is planned.

Up to a third of BCC cases are classified as superficial BCC (sBCC) [13]. Topical treatments effective for sBCC include imiquimod, 5-fluorouracil, and photodynamic therapy, with imiquimod considered the most effective [14]. However, the difficulty in differentiating subtypes clinically makes the accurate risk stratification of BCC even more important.

**Table 1:** Recommended margins for high- and low-risk basal cell carcinoma (BCC) in different countries.

Organization	Low-risk BCC margins	High-risk BCC margins	Deep margin
National Cancer Care Network, United States	Surgical excision 4 mm	Mohs or surgical excision ≥4 mm	Not specified
European Dermatology Forum	Surgical excision 3-4 mm	Mohs or surgical excision 5-10 mm	Level of fascia, perichondrium, or periosteum; less deep for superficial lesions
British Association of Dermatology	Surgical excision 4-5 mm	Mohs or surgical excision ≥5 mm; morphoeic BCC >13-15 mm	Subcutaneous fat (through)
Cancer Council Australia	Surgical excision or Mohs 2-3 mm	Surgical excision or Mohs 3-5 mm	Subcutaneous fat (include)
Sweden National Guidelines	Surgical excision ≥3-4 mm	Surgical excision ≥5 mm	Not specified

## Methods

This paper presents a perspective from a skin cancer expert and a well-established clinic specializing in the treatment of both simple and complex cases of BCC. It addresses the key challenges associated with accurate risk stratification prior to any treatment or procedure. Different immunohistochemical and angiogenic markers for high-risk BCC were also reviewed.

What biomarkers can we use to identify high-risk BCC prior to excisional surgery? Dermatoscopy has improved BCC diagnosis, but the sensitivity and specificity of high-risk BCC signs are not sufficient for accurate diagnosis. For example, the superficial subtype of BCC can be identified in about 80% of cases [15], but this level of accuracy does not apply to specific subtypes of high-risk BCC. A literature review was conducted to identify potential histological markers, and below, the immunohistochemical and angiogenic agent expressions that show promise as BCC risk evaluators are discussed.

## Results

The immunohistochemical and angiogenic markers for BCC risk evaluation are as follows:

### Vascular Endothelial Growth Factor

Vascular endothelial growth factor (VEGF) is implicated in the development of nonmelanoma skin cancers, such as BCC, because keratinocytes respond directly to VEGF, which in turn affects skin carcinogenesis by altering the proliferation and survival of these cells [16]. Studies on the vascular and angiogenic patterns of BCC reveal that the lowest VEGF expression is found in sBCC, while infiltrative and metatypical subtypes exhibit the highest values [17].

### Interleukin-6

It has been known that interleukin-6 (IL-6) concentrations are significantly higher in BCC tumor microenvironments compared to other skin cancers, such as squamous cell cancers [18]. Mawardi et al [19] examined both VEGF-A and IL-6 and discovered a strong correlation with high-risk BCC. They found that a strong VEGF-A expression was significantly more frequent in high-risk aggressive BCC compared to nonaggressive BCC, and IL-6 levels were also indicative of risk (Table 2).

**Table 2:** Vascular endothelial growth factor A (VEGF-A) and interleukin-6 (IL-6) expressions in basal cell carcinoma (BCC) punch biopsy samples from head and neck [19].

Agent expression	Aggressive BCC	Nonaggressive BCC	*P* value
VEGF-A	16	3	<.003
IL-6	13	2	<.001

### Mast Cells

The study by Mawardi et al [19] found that the highest number of mast cells were in the micronodular BCC subtype—a subtype prone to recurrence—and there was a significant difference in mast cell numbers between high-risk aggressive BCC and nonaggressive BCC. Although some authors have suggested that mast cells may not be strictly classifiable as friends or foes in a tumor setting, dermal mast cells may promote skin carcinogenesis by creating an immunosuppressive microenvironment [20]. Recently, for the first time, mast cell activation was observed noninvasively through staining-free visualization of dermal mast cells using two-photon fluorescence lifetime imaging [21]. If such imaging becomes clinically available, it can enhance our ability to stratify BCC risk more accurately before surgery, even without needing a biopsy.

### Cyclin D1 and Bcl-2

Studies have shown statistically significant differences between nonaggressive (ie, nodular) and aggressive (ie, micronodular and infiltrative) types using both these markers. Cyclin D1 levels were higher in the aggressive group (*P*=.04), while Bcl-2 levels were lower in the aggressive group compared to the nonaggressive group (*P*=.01) [22].

## Discussion

Because BCC is the most common tumor in humans and the global incidence of these tumors is increasing, it is important to stratify the risk. This helps reduce health care costs and spares patients from unnecessary invasive surgical procedures. In this context, identifying immunohistochemical and angiogenic markers, such as VEGF-A, IL-6, Cyclin D1, Bcl-2, and mast cells from punch or shave biopsy samples, can assist in determining the need for surgery or when topical therapies may be more suitable. Noninvasive two-photon tomography, in combination with fluorescence lifetime imaging, has been used to visualize human skin mast cells in vivo using a staining-free method, which also holds promise for future applications.

Visualizing immunohistochemical and angiogenic agents in vivo, with or without staining techniques, hints at a future where BCC risk can be assessed accurately prior to surgery, ultimately leading to more patient-centered treatments.

## Abbreviations

BCC: basal cell carcinoma
IL-6: interleukin-6
sBCC: superficial basal cell carcinoma
VEGF: vascular endothelial growth factor
